# 1-(6-Chloro-2-methyl-4-phenyl-3-quinol­yl)ethanone

**DOI:** 10.1107/S1600536809040306

**Published:** 2009-10-10

**Authors:** Hoong-Kun Fun, Wan-Sin Loh, S. Sarveswari, V. Vijayakumar, B. Palakshi Reddy

**Affiliations:** a X-ray Crystallography Unit, School of Physics, Universiti Sains Malaysia, 11800 USM, Penang, Malaysia; b Organic Chemistry Division, School of Science, VIT University, Vellore 632 014, India

## Abstract

In the title compound, C_18_H_14_ClNO, the quinoline ring system is approximately planar with a maximum devation of 0.022 (1) Å and forms a dihedral angle of 62.70 (3)° with the phenyl ring. In the crystal, pairs of C—H⋯O inter­molecular hydrogen bonds link neighbouring mol­ecules into inversion dimers, forming *R*_2_^2^(14) ring motifs. These inversion dimers are stacked along the *b* axis. The structure is further stabilized by C—H⋯π inter­actions.

## Related literature

For reference bond-length data, see: Allen *et al.* (1987 ▶). For background to quinolines, see: Morimoto *et al.* (1991 ▶); Michael (1997 ▶); Markees *et al.* (1970 ▶); Campbell *et al.* (1988 ▶); Maguire *et al.* (1994 ▶); Kalluraya & Sreenivasa (1998 ▶); Roma *et al.* (2000 ▶); Chen *et al.* (2001 ▶); Skraup (1880 ▶); Katritzky & Arend (1998 ▶); Jiang & Si (2002 ▶). For the biological activity of chalcones, see: Dimmock *et al.* (1999 ▶); Yamazaki *et al.* (2002 ▶). For a related structure, see: Fun *et al.* (2009 ▶). For hydrogen-bond motifs, see: Bernstein *et al.* (1995 ▶). For the stability of the temperature controller used for the data collection, see: Cosier & Glazer (1986 ▶).
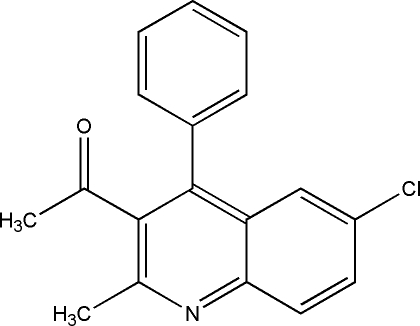


## Experimental

### 

#### Crystal data

C_18_H_14_ClNO
*M* *_r_* = 295.75
Monoclinic, 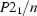

*a* = 10.4633 (2) Å
*b* = 7.7959 (1) Å
*c* = 17.5925 (3) Å
β = 90.887 (1)°
*V* = 1434.86 (4) Å^3^
*Z* = 4
Mo *K*α radiation
μ = 0.26 mm^−1^
*T* = 100 K
0.57 × 0.34 × 0.27 mm

#### Data collection

Bruker SMART APEXII CCD area-detector diffractometer
Absorption correction: multi-scan (**SADABS**; Bruker, 2005 ▶) *T*_min_ = 0.865, *T*_max_ = 0.932
32340 measured reflections
7613 independent reflections
6588 reflections with *I* > 2σ(*I*)
*R*_int_ = 0.023

#### Refinement

*R*[*F* ^2^ > 2σ(*F* ^2^)] = 0.036
*wR*(*F* ^2^) = 0.107
*S* = 1.07
7613 reflections
192 parameters
H-atom parameters constrained
Δρ_max_ = 0.58 e Å^−3^
Δρ_min_ = −0.24 e Å^−3^

### 

Data collection: *APEX2* (Bruker, 2005 ▶); cell refinement: *SAINT* (Bruker, 2005 ▶); data reduction: *SAINT*; program(s) used to solve structure: *SHELXTL* (Sheldrick, 2008 ▶); program(s) used to refine structure: *SHELXTL*; molecular graphics: *SHELXTL* software used to prepare material for publication: *SHELXTL* and *PLATON* (Spek, 2009 ▶).

## Supplementary Material

Crystal structure: contains datablocks global, I. DOI: 10.1107/S1600536809040306/wn2352sup1.cif

Structure factors: contains datablocks I. DOI: 10.1107/S1600536809040306/wn2352Isup2.hkl

Additional supplementary materials:  crystallographic information; 3D view; checkCIF report

## Figures and Tables

**Table 1:** Hydrogen-bond geometry (Å, °)

*D*—H⋯*A*	*D*—H	H⋯*A*	*D*⋯*A*	*D*—H⋯*A*
C15—H15*A*⋯O1^i^	0.93	2.55	3.2047 (10)	128
C11—H11*A*⋯*Cg*1^ii^	0.93	2.78	3.6416 (7)	155
C13—H13*A*⋯*Cg*2^iii^	0.93	2.92	3.6255 (8)	133

Symmetry codes: (i) 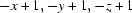; (ii) 
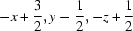
; (iii) 
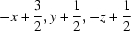
. *Cg*1 and *Cg*2 are the centroids of the C1–C9/N1 and C10–C15 ring systems, respectively.

**Fig. 1.**
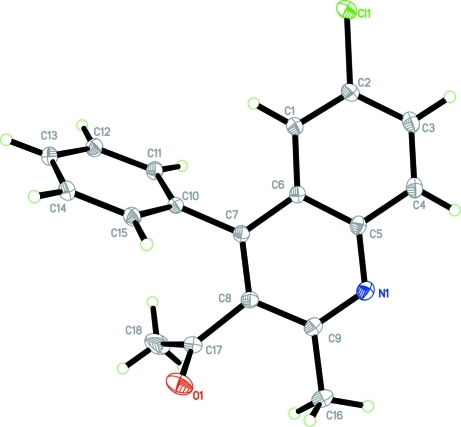
The molecular structure of the title compound, showing 50% probability displacement ellipsoids and the atom-numbering scheme.

**Fig. 2.**
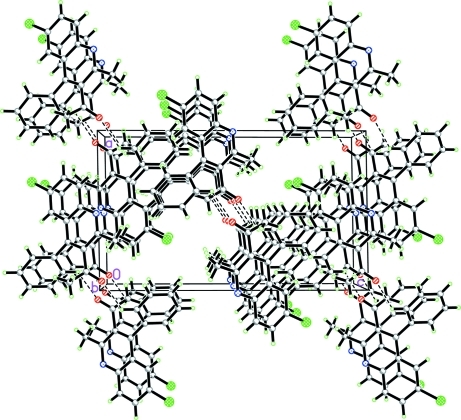
The crystal packing of the title compound, viewed along the b axis, showing the R22(14) ring motifs. C—H···O intermolecular interactions are shown as dashed lines.

## supplementary crystallographic information

### Comment

Quinolines and their derivatives are very important compounds because of their wide occurrence in natural products (Morimoto *et al.*, 1991; Michael, 1997) and biologically active compounds (Markees *et al.*, 1970; Campbell *et al.*, 1988). A large variety of quinolines have interesting physiological activities and have found attractive applications as pharmaceuticals, agrochemicals and as synthetic building blocks (Maguire *et al.*, 1994; Kalluraya & Sreenivasa, 1998; Roma *et al.*, 2000; Chen *et al.*, 2001; Skraup, 1880). Many synthetic methods such as the Skraup, Doebner-Von Miller, Friedländer and Combes reactions have been developed for the preparation of quinolines, but due to their great importance, the synthesis of new derivatives of quinoline remains an active research area (Katritzky & Arend, 1998; Jiang & Si, 2002). Chalcones are open-chain flavonoids, possessing a variety of biological activities, including antioxidant, anti-inflammatory, antimicrobial, antiprotozoal, antiulcer, as well as other activities (Dimmock *et al.*, 1999). More importantly, chalcones have shown several anticancer activities, such as inhibitors of cancer cell proliferation, carcinogenesis, and metastasis (Yamazaki *et al.*, 2002).

In the crystal structure (Fig. 1), bond lengths (Allen *et al.*, 1987) and angles are within normal ranges and comparable to those in a closely related structure (Fun *et al.*, 2009). The quinoline ring system (C1–C9/N1) is approximately planar, with a maximum devation of 0.022 (1) Å at atom C1. The phenyl ring (C10–C15) forms a dihedral angle of 62.70 (3)° with the mean plane of the quinoline ring system. In the crystal packing (Fig. 2), pairs of C15—H15A···O1 hydrogen bonds link neighbouring molecules into dimers, forming *R*_2_^2^(14) ring motifs (Bernstein *et al.*, 1995). These inversion dimers are stacked along the *b* axis. The crystal structure is further stabilized by C—H···π interactions (Table 1), involving the C1–C9/N1 (centroid *Cg*1) and C10–C15 (centroid *Cg*2) ring systems.

### Experimental

A mixture of 2-amino-5-chlorobenzophenone (2.3 g, 0.01 mol) and acetylacetone (1.0 g, 0.01 mol) with 0.15 ml concentrated HCl in a beaker was subjected to microwave irradiation for about 6 min. After completion of the reaction (monitored by TLC), the reaction mixture was washed with saturated solvent NaHCO_3_ (10 ml) and then it was dried. After that it was washed with petroleum ether and recrystallized with chloroform (*M. p.* 224–226°C). IR (cm^-1^): 1704, 1480, 1385, 840, 711.

### Refinement

All  H atoms were positioned geometrically [C—H = 0.93 or 0.96 Å] and were refined using a riding model, with *U*_iso_(H) = 1.2*U*_eq_(Csp^2^) or 1.5*U*_eq_(methyl C). A rotating-group model was applied for the methyl groups.

### Crystal data

**Table:**

C_18_H_14_ClNO	*F*(000) = 616
*M**_r_* = 295.75	*D*_x_ = 1.369 Mg m^−^^3^
Monoclinic, *P*2_1_/*n*	Mo *K*α radiation, λ = 0.71073 Å
Hall symbol: -P 2yn	Cell parameters from 9994 reflections
*a* = 10.4633 (2) Å	θ = 2.3–37.6°
*b* = 7.7959 (1) Å	µ = 0.26 mm^−^^1^
*c* = 17.5925 (3) Å	*T* = 100 K
β = 90.887 (1)°	Block, yellow
*V* = 1434.86 (4) Å^3^	0.57 × 0.34 × 0.27 mm
*Z* = 4	

### Data collection

**Table:**

Bruker SMART APEXII CCD area-detector diffractometer	7613 independent reflections
Radiation source: fine-focus sealed tube	6588 reflections with *I* > 2σ(*I*)
graphite	*R*_int_ = 0.023
φ and ω scans	θ_max_ = 37.6°, θ_min_ = 2.3°
Absorption correction: multi-scan (*SADABS*; Bruker, 2005)	*h* = −17→16
*T*_min_ = 0.865, *T*_max_ = 0.932	*k* = −13→12
32340 measured reflections	*l* = −30→30

### Refinement

**Table:**

Refinement on *F*^2^	Primary atom site location: structure-invariant direct methods
Least-squares matrix: full	Secondary atom site location: difference Fourier map
*R*[*F*^2^ > 2σ(*F*^2^)] = 0.036	Hydrogen site location: inferred from neighbouring sites
*wR*(*F*^2^) = 0.107	H-atom parameters constrained
*S* = 1.07	*w* = 1/[σ^2^(*F*_o_^2^) + (0.0574*P*)^2^ + 0.2858*P*] where *P* = (*F*_o_^2^ + 2*F*_c_^2^)/3
7613 reflections	(Δ/σ)_max_ < 0.001
192 parameters	Δρ_max_ = 0.58 e Å^−^^3^
0 restraints	Δρ_min_ = −0.24 e Å^−^^3^

### Special details

**Table:**

Experimental. The crystal was placed in the cold stream of an Oxford Cyrosystems Cobra open-flow nitrogen cryostat (Cosier & Glazer, 1986) operating at 100.0 (1) K.
Geometry. All e.s.d.'s (except the e.s.d. in the dihedral angle between two l.s. planes) are estimated using the full covariance matrix. The cell e.s.d.'s are taken into account individually in the estimation of e.s.d.'s in distances, angles and torsion angles; correlations between e.s.d.'s in cell parameters are only used when they are defined by crystal symmetry. An approximate (isotropic) treatment of cell e.s.d.'s is used for estimating e.s.d.'s involving l.s. planes.
Refinement. Refinement of *F*^2^ against ALL reflections. The weighted *R*-factor *wR* and goodness of fit *S* are based on *F*^2^, conventional *R*-factors *R* are based on *F*, with *F* set to zero for negative *F*^2^. The threshold expression of *F*^2^ > σ(*F*^2^) is used only for calculating *R*-factors(gt) *etc*. and is not relevant to the choice of reflections for refinement. *R*-factors based on *F*^2^ are statistically about twice as large as those based on *F*, and *R*- factors based on ALL data will be even larger.

### Fractional atomic coordinates and isotropic or equivalent isotropic displacement parameters (Å^2^)

**Table:**

	*x*	*y*	*z*	*U*_iso_*/*U*_eq_	
Cl1	1.179322 (16)	0.65521 (3)	0.247848 (10)	0.02152 (5)	
O1	0.56817 (6)	0.27543 (9)	0.51440 (4)	0.02585 (12)	
N1	0.98196 (6)	0.23575 (8)	0.49910 (3)	0.01532 (10)	
C1	0.98802 (6)	0.50843 (9)	0.32517 (4)	0.01414 (10)	
H1A	0.9336	0.5589	0.2894	0.017*	
C2	1.11760 (6)	0.53229 (9)	0.32124 (4)	0.01548 (11)	
C3	1.20321 (6)	0.46250 (9)	0.37595 (4)	0.01719 (11)	
H3A	1.2907	0.4809	0.3723	0.021*	
C4	1.15518 (6)	0.36706 (9)	0.43465 (4)	0.01614 (11)	
H4A	1.2107	0.3226	0.4714	0.019*	
C5	1.02224 (6)	0.33535 (8)	0.43995 (4)	0.01347 (10)	
C6	0.93765 (6)	0.40614 (8)	0.38437 (3)	0.01243 (10)	
C7	0.80451 (6)	0.36943 (8)	0.39077 (3)	0.01238 (10)	
C8	0.76613 (6)	0.26872 (8)	0.45085 (3)	0.01334 (10)	
C9	0.85868 (6)	0.20413 (9)	0.50445 (4)	0.01465 (10)	
C10	0.70864 (6)	0.44156 (8)	0.33610 (3)	0.01260 (10)	
C11	0.70861 (6)	0.39520 (9)	0.25902 (4)	0.01491 (10)	
H11A	0.7714	0.3219	0.2410	0.018*	
C12	0.61476 (6)	0.45857 (9)	0.20938 (4)	0.01612 (11)	
H12A	0.6143	0.4263	0.1585	0.019*	
C13	0.52150 (6)	0.57038 (9)	0.23602 (4)	0.01573 (11)	
H13A	0.4589	0.6126	0.2029	0.019*	
C14	0.52217 (6)	0.61880 (9)	0.31229 (4)	0.01545 (11)	
H14A	0.4607	0.6947	0.3298	0.019*	
C15	0.61476 (6)	0.55367 (9)	0.36233 (4)	0.01429 (10)	
H15A	0.6141	0.5849	0.4133	0.017*	
C16	0.81736 (8)	0.09262 (10)	0.56922 (4)	0.02031 (13)	
H16B	0.8914	0.0471	0.5951	0.030*	
H16C	0.7660	−0.0001	0.5499	0.030*	
H16A	0.7683	0.1595	0.6041	0.030*	
C17	0.62761 (6)	0.22144 (9)	0.46068 (4)	0.01645 (11)	
C18	0.56993 (9)	0.09744 (14)	0.40461 (5)	0.02852 (17)	
H18A	0.4785	0.1000	0.4083	0.043*	
H18B	0.6004	−0.0162	0.4156	0.043*	
H18C	0.5940	0.1292	0.3541	0.043*	

### Atomic displacement parameters (Å^2^)

**Table:**

	*U*^11^	*U*^22^	*U*^33^	*U*^12^	*U*^13^	*U*^23^
Cl1	0.01532 (7)	0.02794 (10)	0.02141 (8)	−0.00307 (5)	0.00332 (5)	0.00537 (6)
O1	0.0226 (3)	0.0312 (3)	0.0242 (3)	0.0004 (2)	0.0103 (2)	−0.0010 (2)
N1	0.0162 (2)	0.0145 (2)	0.0153 (2)	0.00089 (17)	−0.00179 (17)	0.00089 (17)
C1	0.0122 (2)	0.0158 (2)	0.0145 (2)	0.00001 (19)	0.00039 (18)	0.00054 (19)
C2	0.0132 (2)	0.0166 (3)	0.0167 (2)	−0.00137 (19)	0.00138 (19)	0.0000 (2)
C3	0.0123 (2)	0.0174 (3)	0.0219 (3)	−0.0004 (2)	−0.0010 (2)	−0.0008 (2)
C4	0.0133 (2)	0.0153 (3)	0.0197 (3)	0.00069 (19)	−0.0032 (2)	−0.0008 (2)
C5	0.0136 (2)	0.0125 (2)	0.0143 (2)	0.00098 (18)	−0.00177 (18)	−0.00102 (18)
C6	0.0118 (2)	0.0130 (2)	0.0125 (2)	0.00046 (18)	−0.00022 (17)	−0.00075 (18)
C7	0.0121 (2)	0.0135 (2)	0.0115 (2)	0.00065 (17)	0.00041 (17)	−0.00052 (18)
C8	0.0136 (2)	0.0143 (2)	0.0122 (2)	0.00014 (18)	0.00090 (18)	−0.00009 (18)
C9	0.0168 (2)	0.0135 (2)	0.0136 (2)	0.00065 (19)	−0.00055 (19)	0.00067 (19)
C10	0.0108 (2)	0.0151 (2)	0.0119 (2)	−0.00022 (18)	0.00032 (17)	0.00094 (18)
C11	0.0140 (2)	0.0181 (3)	0.0127 (2)	0.0014 (2)	0.00037 (18)	−0.0009 (2)
C12	0.0151 (2)	0.0200 (3)	0.0132 (2)	−0.0002 (2)	−0.00113 (19)	0.0002 (2)
C13	0.0127 (2)	0.0181 (3)	0.0164 (2)	−0.00090 (19)	−0.00199 (19)	0.0024 (2)
C14	0.0116 (2)	0.0171 (3)	0.0176 (3)	0.00086 (19)	0.00059 (19)	0.0011 (2)
C15	0.0122 (2)	0.0171 (3)	0.0136 (2)	0.00082 (19)	0.00122 (18)	−0.00023 (19)
C16	0.0232 (3)	0.0199 (3)	0.0177 (3)	0.0000 (2)	0.0000 (2)	0.0065 (2)
C17	0.0150 (2)	0.0194 (3)	0.0150 (2)	−0.0010 (2)	0.00195 (19)	0.0031 (2)
C18	0.0256 (4)	0.0377 (5)	0.0222 (3)	−0.0151 (3)	0.0014 (3)	−0.0032 (3)

### Geometric parameters (Å, °)

**Table:**

Cl1—C2	1.7401 (7)	C10—C15	1.3985 (9)
O1—C17	1.2146 (9)	C10—C11	1.4032 (9)
N1—C9	1.3181 (9)	C11—C12	1.3944 (9)
N1—C5	1.3701 (9)	C11—H11A	0.9300
C1—C2	1.3714 (9)	C12—C13	1.3949 (10)
C1—C6	1.4196 (9)	C12—H12A	0.9300
C1—H1A	0.9300	C13—C14	1.3937 (10)
C2—C3	1.4137 (10)	C13—H13A	0.9300
C3—C4	1.3742 (10)	C14—C15	1.3947 (9)
C3—H3A	0.9300	C14—H14A	0.9300
C4—C5	1.4173 (9)	C15—H15A	0.9300
C4—H4A	0.9300	C16—H16B	0.9600
C5—C6	1.4203 (9)	C16—H16C	0.9600
C6—C7	1.4284 (9)	C16—H16A	0.9600
C7—C8	1.3813 (9)	C17—C18	1.5012 (11)
C7—C10	1.4891 (9)	C18—H18A	0.9600
C8—C9	1.4325 (9)	C18—H18B	0.9600
C8—C17	1.5081 (9)	C18—H18C	0.9600
C9—C16	1.5021 (10)		
			
C9—N1—C5	118.23 (6)	C12—C11—C10	120.24 (6)
C2—C1—C6	119.46 (6)	C12—C11—H11A	119.9
C2—C1—H1A	120.3	C10—C11—H11A	119.9
C6—C1—H1A	120.3	C11—C12—C13	120.03 (6)
C1—C2—C3	122.01 (6)	C11—C12—H12A	120.0
C1—C2—Cl1	119.39 (5)	C13—C12—H12A	120.0
C3—C2—Cl1	118.59 (5)	C14—C13—C12	119.97 (6)
C4—C3—C2	119.00 (6)	C14—C13—H13A	120.0
C4—C3—H3A	120.5	C12—C13—H13A	120.0
C2—C3—H3A	120.5	C13—C14—C15	120.14 (6)
C3—C4—C5	120.97 (6)	C13—C14—H14A	119.9
C3—C4—H4A	119.5	C15—C14—H14A	119.9
C5—C4—H4A	119.5	C14—C15—C10	120.25 (6)
N1—C5—C4	117.56 (6)	C14—C15—H15A	119.9
N1—C5—C6	123.19 (6)	C10—C15—H15A	119.9
C4—C5—C6	119.25 (6)	C9—C16—H16B	109.5
C1—C6—C5	119.27 (6)	C9—C16—H16C	109.5
C1—C6—C7	122.95 (6)	H16B—C16—H16C	109.5
C5—C6—C7	117.78 (6)	C9—C16—H16A	109.5
C8—C7—C6	118.01 (6)	H16B—C16—H16A	109.5
C8—C7—C10	120.53 (5)	H16C—C16—H16A	109.5
C6—C7—C10	121.43 (5)	O1—C17—C18	121.94 (7)
C7—C8—C9	120.13 (6)	O1—C17—C8	120.60 (7)
C7—C8—C17	121.19 (6)	C18—C17—C8	117.34 (6)
C9—C8—C17	118.67 (6)	C17—C18—H18A	109.5
N1—C9—C8	122.66 (6)	C17—C18—H18B	109.5
N1—C9—C16	117.12 (6)	H18A—C18—H18B	109.5
C8—C9—C16	120.21 (6)	C17—C18—H18C	109.5
C15—C10—C11	119.35 (6)	H18A—C18—H18C	109.5
C15—C10—C7	119.49 (5)	H18B—C18—H18C	109.5
C11—C10—C7	121.13 (6)		
			
C6—C1—C2—C3	−2.09 (10)	C5—N1—C9—C8	0.38 (10)
C6—C1—C2—Cl1	179.09 (5)	C5—N1—C9—C16	179.18 (6)
C1—C2—C3—C4	0.36 (11)	C7—C8—C9—N1	−0.40 (10)
Cl1—C2—C3—C4	179.19 (5)	C17—C8—C9—N1	178.37 (6)
C2—C3—C4—C5	1.27 (10)	C7—C8—C9—C16	−179.17 (6)
C9—N1—C5—C4	−179.52 (6)	C17—C8—C9—C16	−0.40 (9)
C9—N1—C5—C6	0.11 (10)	C8—C7—C10—C15	61.18 (9)
C3—C4—C5—N1	178.51 (6)	C6—C7—C10—C15	−116.81 (7)
C3—C4—C5—C6	−1.13 (10)	C8—C7—C10—C11	−116.99 (7)
C2—C1—C6—C5	2.18 (10)	C6—C7—C10—C11	65.01 (9)
C2—C1—C6—C7	−177.46 (6)	C15—C10—C11—C12	−0.88 (10)
N1—C5—C6—C1	179.78 (6)	C7—C10—C11—C12	177.30 (6)
C4—C5—C6—C1	−0.60 (9)	C10—C11—C12—C13	0.91 (10)
N1—C5—C6—C7	−0.57 (9)	C11—C12—C13—C14	0.02 (10)
C4—C5—C6—C7	179.06 (6)	C12—C13—C14—C15	−0.97 (10)
C1—C6—C7—C8	−179.83 (6)	C13—C14—C15—C10	1.00 (10)
C5—C6—C7—C8	0.52 (9)	C11—C10—C15—C14	−0.07 (10)
C1—C6—C7—C10	−1.79 (10)	C7—C10—C15—C14	−178.28 (6)
C5—C6—C7—C10	178.56 (6)	C7—C8—C17—O1	−113.46 (8)
C6—C7—C8—C9	−0.08 (9)	C9—C8—C17—O1	67.78 (9)
C10—C7—C8—C9	−178.14 (6)	C7—C8—C17—C18	70.31 (9)
C6—C7—C8—C17	−178.81 (6)	C9—C8—C17—C18	−108.45 (8)
C10—C7—C8—C17	3.13 (9)		

### Hydrogen-bond geometry (Å, °)

**Table:**

*D*—H···*A*	*D*—H	H···*A*	*D*···*A*	*D*—H···*A*
C15—H15A···O1^i^	0.93	2.55	3.2047 (10)	128
C11—H11A···Cg1^ii^	0.93	2.78	3.6416 (7)	155
C13—H13A···Cg2^iii^	0.93	2.92	3.6255 (8)	133

Symmetry codes: (i) −*x*+1, −*y*+1, −*z*+1; (ii) −*x*+3/2, *y*−1/2, −*z*+1/2; (iii) −*x*+3/2, *y*+1/2, −*z*+1/2.
